# Dovitinib Triggers Apoptosis and Autophagic Cell Death by Targeting SHP-1/*p*-STAT3 Signaling in Human Breast Cancers

**DOI:** 10.1155/2019/2024648

**Published:** 2019-08-14

**Authors:** Yi-Han Chiu, Yi-Yen Lee, Kuo-Chin Huang, Cheng-Chi Liu, Chen-Si Lin

**Affiliations:** ^1^Department of Nursing, St. Mary's Junior College of Medicine, Nursing and Management, Yilan 26647, Taiwan; ^2^Institute of Long-Term Care, Mackay Medical College, New Taipei City 25245, Taiwan; ^3^Department of Veterinary Medicine, School of Veterinary Medicine, National Taiwan University, 1 Sec 4 Roosevelt Road, Taipei 10617, Taiwan; ^4^Holistic Education Center, Mackay Medical College, New Taipei City 25245, Taiwan; ^5^Animal Cancer Center, College of Bioresources and Agriculture, National Taiwan University, 1 Sec 4 Roosevelt Road, Taipei 10617, Taiwan

## Abstract

Breast cancer is the most common cancer and the leading cause of cancer deaths in women worldwide. The rising incidence rate and female mortality make it a significant public health concern in recent years. Dovitinib is a novel multitarget receptor tyrosine kinase inhibitor, which has been enrolled in several clinical trials in different cancers. However, its antitumor efficacy has not been well determined in breast cancers. Our results demonstrated that dovitinib showed significant antitumor activity in human breast cancer cell lines with dose- and time-dependent manners. Downregulation of phosphor-(*p*)-STAT3 and its subsequent effectors Mcl-1 and cyclin D1 was responsible for this drug effect. Ectopic expression of STAT3 rescued the breast cancer cells from cell apoptosis induced by dovitinib. Moreover, SHP-1 inhibitor reversed the downregulation of *p*-STAT3 induced by dovitinib, indicating that SHP-1 mediated the STAT3 inhibition effect of dovitinib. In addition to apoptosis, we found for the first time that dovitinib also activated autophagy to promote cell death in breast cancer cells. In conclusion, dovitinib induced both apoptosis and autophagy to block the growth of breast cancer cells by regulating the SHP-1-dependent STAT3 inhibition.

## 1. Introduction

Breast cancer is the most common cancer in women, and the mortality rate is in the rank of top five cancers [1]. In Taiwan, breast cancer is also the top diagnosed cancer in women. The incidence rate of breast cancer keeps climbing high in the last 30 years. Therefore, breast cancer is the critical public health issue in recent years. It was found that epidermal growth factor receptor- (EGFR-) positive breast cancer was more prevalent in Asian women diagnosed than Western women [2] and high expression of EGFR oncoprotein was associated with advanced stages of breast cancer [3]. The receptors of EGFR family regulate the transcription of molecules that control several cellular functions, including cell proliferation, differentiation, apoptosis, invasion, and angiogenesis [4]. Thus, EGFR is one of the first identified important targets of these novel anti-breast tumor agents in Asia. However, treatment of EGFR inhibitors on breast patients has been found to rapidly advance to resistance and disease progression [5, 6], implying that more effective therapeutic receptor tyrosine kinases (RTKs) inhibitors are required.

Fibroblast growth factors (FGFs) and FGF receptors (FGFRs) signaling network play essential roles to promote angiogenesis and tumor growth by binding to tyrosine kinase [7]. FGFR is reported to be overexpressed and potentially promote tumor growth and invasion in patients with breast cancer [8]. Recent studies reported that FGFR-dependent signaling contributes to a mechanism for intrinsic resistance to EGFR inhibitors in EGFR-dependent cell lines [9, 10]. Taken together, FGFR inhibitors are considered one of the potential RTK inhibitors that can be used to treat patients with breast cancer.

Dovitinib (TKI258) is a small-molecule tyrosine kinase inhibitor targeting multiple RTKs, such as FGFRs [11], vascular endothelial growth factor receptor (VEGFR) [12], fetal liver tyrosine kinase receptor 3 (FLT-3) [13], and colony-stimulating factor receptor 1 (c-Fms) [14], which participates in tumor growth, survival, angiogenesis, and vascular development and is under clinical investigation in different malignancies [15]. According to previous studies, dovitinib exhibits potent tumor growth inhibition in a board range of preclinical animal models and clinical trials, including leukemia, advanced melanoma, endometrial cancer, brain neoplasm, digestive system neoplasm, breast cancer, etc. For example, dovitinib has been shown to have the antitumor effect in endometrial cancer beyond FGFR2-mutated cases [16]. In addition, a preclinical FGFR1-amplified xenograft model demonstrated that dovitinib showed antitumor activity in FGFR-amplified breast cancer cell lines [17]. Moreover, a phase I/II dose-escalation study revealed that dovitinib exhibited an acceptable safety profile at a dose of 400 mg/day and showed clinical benefit by specifically inhibiting FGFR and VEGFR in patients with advanced melanoma [18].

Although several studies have focused on the clinical efficacy of dovitinib in different cancers, comparatively few reports have looked at molecular mechanisms of dovitinib action in cancer cells, especially in breast cancer. In some human tumor models, dovitinib was shown to inhibit the STAT3/5, MAPK, PI3K/AKT/mTOR, and Wnt signaling pathways [19–21]. *In vivo* studies using both Huh-7 and PLC5 xenograft tumors model showed dovitinib downregulated phospho‐(*p*)STAT3 and subsequently reduced the expressions of its downstream-regulated proteins, Mcl-1, survivin, and cyclin D1 [22]. STAT3 plays a vital role in transcriptional regulation of genes involved in cell proliferation and tumor progression triggered by cytokines and growth factors such as EGFR and FGFR [23]. Many protein families act as negative regulators of the STAT3 signaling pathway, such as SH2-domain-containing cytosolic phosphatases, SHP-1 and SHP-2 [24]. SHP-1 belongs to a family of nonreceptor protein tyrosine phosphatases (PTP) and consists of 2 SH2 domains that bind phosphotyrosine, a catalytic PTP domain and a C-terminal tail [25]. Recently, studies identified that dovitinib acts as a SHP-1 agonist or SHP-1 mediator that enhances activation of protein tyrosine phosphatase SHP‐1 and subsequent dephosphorylation of *p*-STAT3^TYR705^, resulting in the downregulation of antiapoptotic STAT3 target genes Mcl-1 and survivin and cyclin D1. However, dovitinib frequently reduced the activity of these signaling pathways while tyrosine kinase receptor‐independent mechanisms of dovitinib also occur.

The mechanism of how breast tumor-suppressive role of dovitinib works is not fully known. Moreover, it is unclear whether dovitinib modulation using a pharmacologically relevant approach would yield similar activation of SHP‐1 and subsequent inhibition of *p*-STAT3^Tyr705^ in breast cancer cells. Here, we report that dovitinib-mediated breast cancer cell death in both autophagic and apoptotic ways. To better understand the molecular mechanism of dovitinib in breast cancer therapy, we investigated the molecular events altered by dovitinib treatment in various breast cancer cells. The role of SHP-1 activity-mediated downregulation of *p*-STAT3 was also confirmed, thus providing novel mechanistic insight into this molecular target for breast cancer.

## 2. Materials and Methods

### 2.1. Reagents and Antibodies

Dovitinib (TKI258) was kindly provided by Novartis Pharmaceuticals. Bafilomycin A1 was purchased from Invivogen (California, USA). Thiazolyl blue tetrazolium bromide (3-(4,5-dimethylthiazol-2-yl)-2,5-diphe- nyltetrazolium bromide, MTT) and acridine orange were purchased from Sigma-Aldrich (Missouri, USA). SHP-1 inhibitor, the STAT3-specific inhibitor, was purchased from Merck Millipore (Massachusetts, USA). G418, being used for selecting transformed with STAT3 plasmid cell line, was purchased from Amresco (Ohio, USA). Antibody for immunoblotting, such as PARP, was purchased from Santa Cruz (Dallas, USA). Other antibodies, such as beclin 1, cyclin D1, Mcl-1, survivin, *p*-STAT3^Tyr705^, STAT3, SQSTM1/*p*62, and SHP-1, were from Cell Signaling (Massachusetts, USA).

### 2.2. Cell Culture

The MCF-7, HCC1937, MDA-MB-231, MDA-MB-468, MDA-MB-453, and SK-BR-3 cell lines were acquired from American Type Culture Collection (Virginia, USA). The MDA-MB-468 with STAT3 overexpression cell line was generously provided by Dr. Liu CY, working in Division of Hematology and Oncology, Department of Medicine, Taipei Veterans General Hospital (Taipei, Taiwan). All cell lines were immediately expanded and frozen down immediately after acquiring. All cell lines could be restarted every 3 months from a frozen vial of the same batch of cells. Cells except for MDA-MB-468 with STAT3 overexpression were maintained as described culture medium by ATCC; MDA-MB-468 with STAT3 overexpression cells was maintained in L-15 medium with G418 700 *μ*g/mL. All media were supplemented with 10% FBS (Caisson, USA), 100 units/mL penicillin, 100 mg/mL streptomycin, and 25 mg/mL amphotericin B (Caisson, USA). All human breast cancer cell lines were incubated in a humidified incubator at 37°C in an atmosphere of 5% CO_2_ in air.

### 2.3. Cell Viability Analysis

The effect of individual test agents on cell viability was assessed by using the thiazolyl blue tetrazolium bromide (MTT). Human breast cancer cells were seeded in the density of 3,000 cells/well with 200 *μ*L FBS-contained cultured medium in 96-well flat-bottom plate and incubated under 37°C and 5% CO_2_ for 24 hours. The very next day, the medium with FBS was removed and 200 *μ*L serum-free medium with various concentrations of dovitinib was added and dissolved in DMSO in serum-free medium, and human breast cancer cells were cocultured with dovitinib under 37°C and 5% CO_2_ for different time intervals. Controls received DMSO vehicle at a concentration equal to that in the highest dosage of drug-treated cells. After coculturing with dovitinib for a period of time, 20 *μ*L of 0.5 mg/mL MTT (1/10 volume of the medium) was added and further incubated under 37°C and 5% CO_2_ for 3 more hours. At the end of the incubation period, the medium was removed and 200 *μ*L DMSO was added and then incubated in no-light condition at room temperature for 15 minutes with a gentle shake. After the incubation period, the 96-well plate was measured at a wavelength of 570 nm with background subtraction at 690 nm by using SpectraMax M5 multimode microplate readers (Molecular Devices, USA).

### 2.4. Autophagy Analysis

The following two methods were used to assess drug-induced autophagy: western blot analysis of microtubule-associated protein-1 light chain 3 (LC3 II) and immunoﬂuorescence of acridine orange. Formation of acidic vesicular organelles (AVOs), a morphological characteristic of autophagy, was detected by acridine orange staining [26]. Cells were stained with 5 mg/mL acridine orange for 10 min at room temperature, and samples were observed under a Nikon Eclipse TS100-F ﬂuorescence microscope (Nikon, Japan).

To quantify the percentage of cells with acidic vacuolar organelles (red-marked cells), human breast cancer cells treated with the indicated concentration of dovitinib were stained with acridine orange and incubated for 10 min in the dark at room temperature. The percentage of autophagic cells (containing the red-marked organelle in the cytoplasm) was analyzed with a FASCaliber flow cytometer.

### 2.5. Apoptosis Analysis

The following two methods were used to assess dovitinib-induced apoptotic cell death: measurement of apoptotic cells by flow cytometry (sub-G1) and western blot analysis for PARP caspases cleavage. For measurement of sub-G1 percentage, human breast cancer cells were treated with DMSO or dovitinib at the indicated dose for 24 hours. The human breast cancer cells were harvested and washed with ice-cold phosphate-buffered saline (PBS) solution twice. They were vortexed gently, and the ice-cold 70% EtOH was added for fixation of the sample lysate at the same time. They were stored at −20°C in a refrigerator for at least 1 day. The pellets were resuspended in PBS and then washed with PBS twice. Samples were incubated with 10 *μ*g/mL DNase-free RNase A (Sigma-Aldrich, USA) and 83 *μ*g/mL propidium iodide (Sigma-Aldrich, USA) at 37°C for 30 minutes. The percentage of apoptotic cells was shown by cell-cycle distribution using flow cytometry. The DNA content of individual cells was analyzed with the fluorescence-activated sorter. Cells with less DNA than that of G1/G0 cells were considered to be apoptotic cells.

### 2.6. Western Blot Analysis

Cell lysates of human breast cancer cells treated with drugs at the indicated concentration for certain periods of time were prepared for immunoblotting of *p*-STAT3, STAT3, cyclinD1, PARP, Mcl-1, survivin, LC3, p62, beclin 1, and *α*-actin. Human breast cancer cells treated with DMSO and other various concentrations of drugs were collected by trypsinization with Trypsin-EDTA solution and washed with ice-cold PBS. Then, the human breast cancer cell pellets were resuspended in 50–60 *μ*L of RIPA lysis buffer (50 mM Tris-HCl (pH 7.4), 0.25% sodium deoxycholate, 1% Nonidet P-40, 150 mM sodium chloride (NaCl), 1 mM EDTA, 1 mM PMSF, 1 mM sodium orthovanadate (Na_3_VO_4_), 1 mM sodium fluoride (NaF), 1.5 *μ*g/ml aprotinin, 1 *μ*g/ml leupeptin and 1 *μ*g/ml pepstatin) for 30 minutes and vortexed gently every 10 minutes. After incubation with RIPA lysis buffer, the physical disruption method was applied for lysis of the remaining pellets. The sonication was proceeded with a Misonix Sonicator S-4000 (New York, USA) as follows: Probe sonication performed to the lysates on ice with 6 cycles of 2-second bursts and 10-second rest at burst amplitude setting of 10. Soluble cell lysates were collected after centrifugation at 200*g* for 20 minutes. The supernatant was collected, and the protein concentrations of the lysates were determined by using a BCA Protein Assay Reagent (Thermo, USA), and the absorbance was measured at 595 nm by using SpectraMax M5 multi-mode microplate readers (Molecular Devices, USA). The lysates were aliquoted with 50 *μ*g/mL in each eppendorf with the sample buffer (0.3 M Tris-HCl, 5% SDS, 50% glycerol, 100 mM dithiothreitol (DTT)) and stored in −80°C refrigerator. Each sample lysate was defrosted and boiled in sample buffer at 100°C for 5–10 minutes before running the gel. The stacking gel (DDW, 30% acrylamide, 1.0 M Tris (pH 6.8), 10% SDS, 10% ammonium persulfate, and TEMED) and 8%/12% resolving gel with DDW, 30% acrylamide, 1.5 M Tris (pH8.8), 10% SDS, 10% ammonium persulfate, and TEMED were prepared. The SDS-PAGE gel was prerun at 80 V for 10 minutes before loading the sample lysates. Equal amounts of protein were loaded into the wells along with molecular weight markers, and the stacking gel and resolving gel were run at 80 V and at 140 V, respectively. Then, the protein from the gel is transferred to the PVDF membrane (Millipore, USA) with the use of wet transfer cell (Bio-rad, USA). The membranes were washed twice with TBS (0.3% (wt/vol) Tris, 0.8% (wt/vol) NaCl, and 0.02% (wt/vol) KCl) containing 0.1% Tween 20 (TBST) and then incubated with TBST containing 5% bovine serum albumin (Sigma, USA) for 1 hour to block nonspecific antibody binding. Then, every PVDF membrane was incubated at 4°C overnight with a primary antibody in TBS containing 5% bovine serum albumin. The membranes were washed twice with TBST and then incubated at room temperature for one hour with horseradish peroxidase- (HRP-) conjugated goat anti-rabbit or anti-mouse immunoglobulin G (IgG) diluted 1 : 10,000 in TBS containing 5% bovine serum albumin at room temperature. The membranes were washed for three times with TBST, and bound antibody was visualized by chemiluminescent HRP substrate (Millipore, USA).

### 2.7. MDA-MB-468 Cells with Ectopic Expression of STAT3

The stable clone cells, MDA-MB-468 with STAT3 overexpression, were prepared for evaluating the major target of dovitinib. MDA-MB-468 with STAT3 overexpression cells were cultured in the presence of G418 (0.7 mg/mL). MDA-MB-468 with STAT3 overexpression cells were treated with the indicated concentration of dovitinib for 24 hours. At the endpoint of treatment, the cell pellets were collected and aliquoted into two parts: one for sub-G1 population analysis and the other for protein immunoblotting analysis.

### 2.8. Statistical Analysis

Data are expressed as individual data or mean ± SD. Experiments were repeated at least three times with similar result. Analysis significance was performed using the Student's t-test (Microsoft Excel), and *P*-value < 0.05 was considered significant.

## 3. Results

### 3.1. Inhibition of Human Breast Cancer Cell Viability by Dovitinib in a Dose- and Time-Dependent Manner

**Figure 1:**
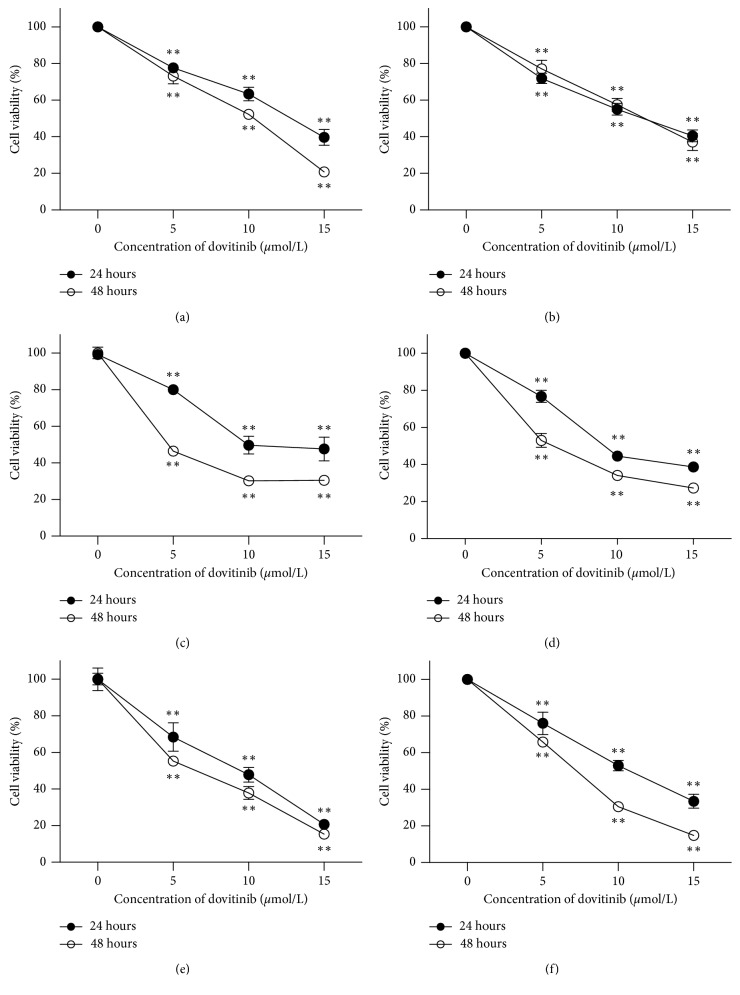
Dovitinib inhibited cell proliferation in a dose- and time-dependent manner. The breast cancer cell lines were treated with dovitinib at the indicated doses for 24 and 48 h, and cell viability was assessed by MTT assay. Points, mean; bars, SD (*N* = 3). ^*∗*^*p* < 0.05; ^*∗∗*^*p* < 0.01. (a) HCC1937. (b) MCF-7. (c) MDA-MB-231. (d) MDA-MB-453. (e) MDA-MB-468. (f) SK-BR-3.

The effect of dovitinib on cell viability in six human breast cancer cell lines (HCC1937, MCF-7, MDA-MB-231, MDA-MB-453, MDA-MB-468, and SK-BR-3) was evaluated for 24 h and 48 h by MTT assays. All the optical density (OD) values of dovitinib-treating groups were compared to the OD values of the control group, in which there was no dovitinib added. Dovitinib decreased the cell numbers in a dose-dependent manner in all tested cell lines (Figure 1), displaying a minor difference of IC_50_. The inhibitory effects were similar in HCC1937 cells (estimated IC_50_, 13.8 ± 2.1 *μ*mole/L), MCF-7 cells (estimated IC_50_, 12.7 ± 3.4 *μ*mole/L), MDA-MB-231 cells (estimated IC_50_, 11.9 ± 3.8 *μ*mole/L), MDA-MB-453 cells (estimated IC_50_, 9.7 ± 1.9 *μ*mole/L), MDA-MB-468 cells (estimated IC_50_, 10.1 ± 2.4 *μ*mole/L), and SK-BR-3 cells (estimated IC_50_, 11.7 ± 2.8 *μ*mole/L). Therefore, the susceptibility of these cancer cells to dovitinib was considered to be similar. In addition, the drug effect was persistent even at 48 hours and the cell numbers reduced much lower than that at 24 hours to reveal that dovitinib inhibited cell growth in a time-dependent manner (Figure 1).

### 3.2. Dovitinib-Mediated Autophagic Cell Death and Induced Accumulation of Autophagic Markers

**Figure 2:**
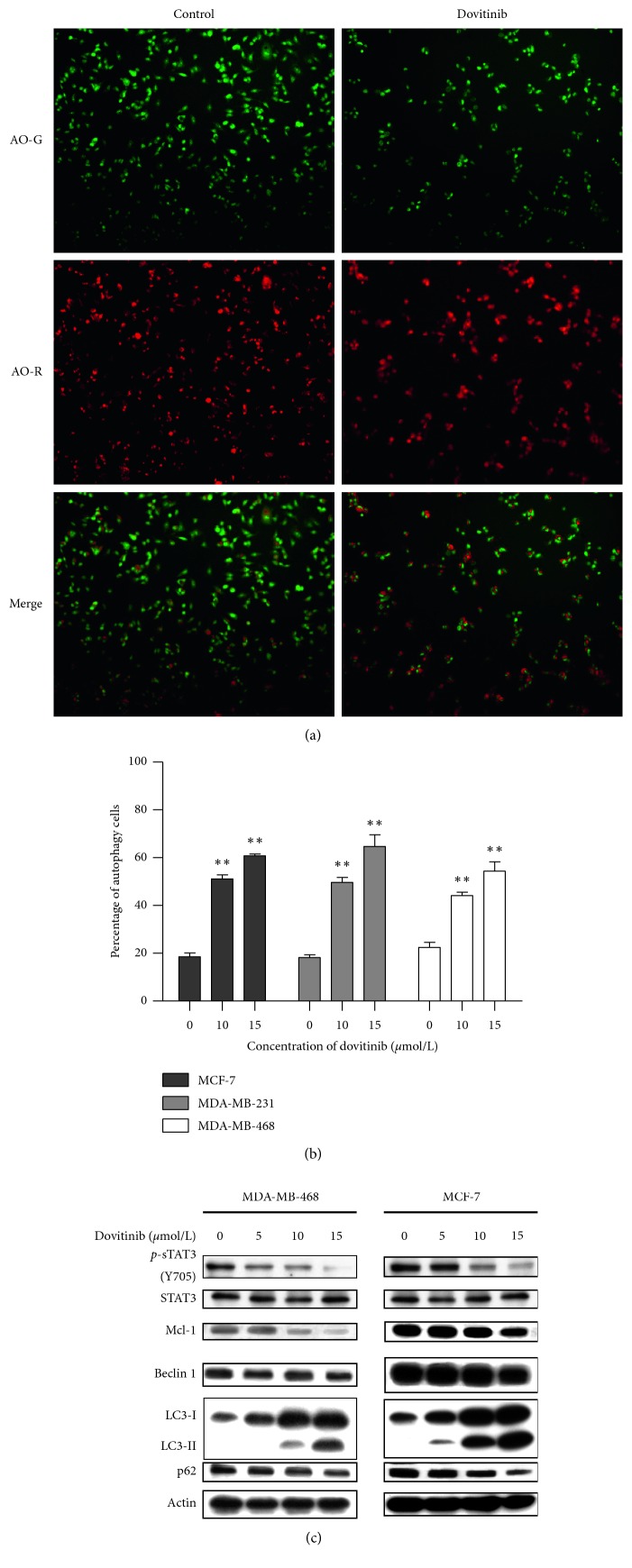
Dovitinib increased the autophagy-mediated cell death in the breast cancer cell. (a) MCF-7 cells were exposed to dovitinib (15 *μ*mole/L) for 24 h. Detection of autophagy was performed by staining the cells with acridine orange for 15 minutes and examined by fluorescence microscopy. AO-R indicated the formation of acidic vesicular organelles. (b) Cells were treated with dovitinib (10 and 15 *μ*mole/L) for 24 h, and the autophagic vacuoles were analyzed by staining of acridine orange. Columns, mean; bars, SD (*N* = 3). ^*∗*^*p* < 0.05; ^*∗∗*^*p* < 0.01. (c) The protein extracts from dovitinib-treated were subjected to immunoblot analysis for *p-*STAT3, STAT3, Mcl-1, beclin1, LC3B, p62, and actin.

Recent studies indicate that chemotherapeutic drugs trigger autophagic but not apoptotic cell death in various cancer cells [27]. The process of autophagy starts with the autophagosome formation and subsequently fuses with an acidic lysosome to form an autolysosome [28]. In order to verify whether dovitinib induced the autophagic pathway, acridine orange staining was employed to visualize acidic vesicular organelles (AO-R positive cells) in control and dovitinib-treated MCF-7 cells. As shown in Figure 2(a), dovitinib treatment markedly elevated the amount of AO-R positive cells, indicating that dovitinib induced a high basal level of autophagic activities. We also evaluated the autophagic cell death by acridine orange staining with flow cytometry in three breast cancer cells treated with 0, 10, and 15 *μ*mole/L dovitinib for 24 h. As shown in Figure 2(b), the percentage of the autophagic cell was 52.1 ± 2.5 and 63.9 ± 1.4% when exposed to 10 and 15 *μ*mole/L dovitinib for 24 h in MCF-7 cells, 49.13 ± 2.6 and 67.2 ± 6.1% in MDA-MB-231 cells, and 47.2 ± 1.6 and 55.4 ± 4.7% in MDA-MB-468 cells. These results indicated that dovitinib induced the autophagy of various breast cancer cells in a dose-dependent manner.

To obtain better insight into the mechanism of dovitinib-induced autophagy, we next analyzed the effects of dovitinib on autophagy-related proteins by western blot analysis. As shown in Figure 2(c), the expression levels of *p-*STAT3, Mcl-1, and beclin 1 was decreased significantly in response to 5, 10, and 15 *μ*mole/L dovitinib in both MDA-MB-468 and MCF-7 breast cell lines. It was reported Mcl-1 could inhibit autophagy by overexpression of beclin 1 [29]. The decreases in the expression levels of Mcl-1 and beclin 1 suggest that Mcl-1 regulates autophagy at least in part by downregulating the activity of beclin-1. We also observed the expression levels of the protein LC3B-I (an unprocessed form of LC3) and the cleaved protein LC3B-II (lipidated and autophagosome-associated form of LC3) were markedly increased in both MDA-MB-468 and MCF-7 breast cell lines following dovitinib treatment at various concentrations compared with the nontreated cells (Figure 2(c)). It was noted that when the expression of Mcl-1 was suppressed, the autophagy markers, LC3-II and p62, also responded to the changing of Mcl-1: LC3-II expression increased and p62 expression decreased (Figure 2(c)). In brief, dovitinib had induced autophagy in breast cancer cells through inhibiting STAT3/Mcl-1 axis and resulted in the formation of autophagy.

### 3.3. Blocking Autophagy Reduced the Antitumor Effects of Dovitinib

**Figure 3:**
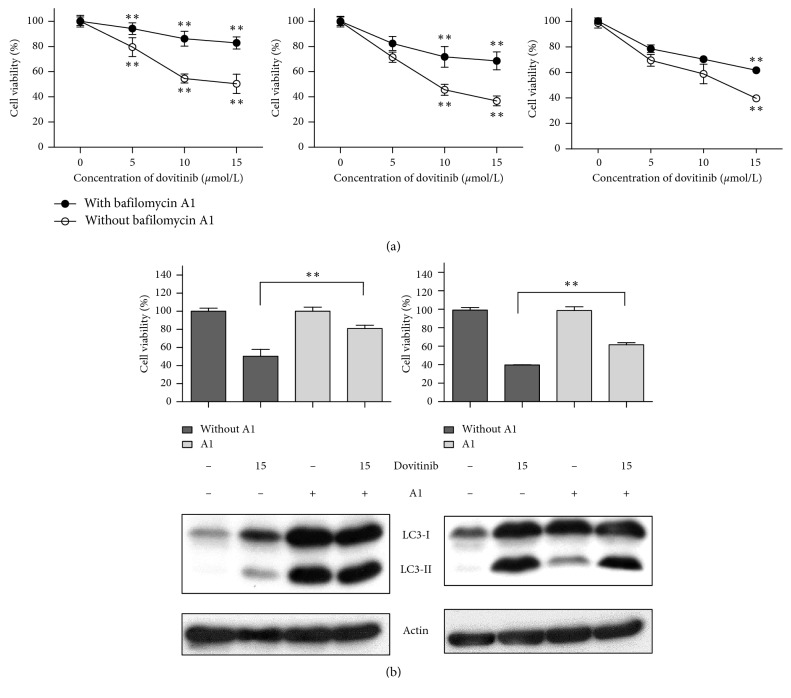
Blocking autophagy reduced the antitumor effects of dovitinib. (a) Cotreatment with the autophagy inhibitor, bafilomycin A1 (20 *μ*mole/L), reduced the effect of dovitinib on cell death. Cells were treated with dovitinib at the indicated doses and/or bafilomycin A1 for 24 h. Cell viability was analyzed by MTT assay. Points, mean; bars, SD (*N* = 3). ^*∗*^*p* < 0.05; ^*∗∗*^*p* < 0.01. (b) MCF-7 cells were treated with dovitinib at 15 *μ*mol/L and/or bafilomycin A1 for 24 h. Cell viability was analyzed by MTT assay. Cell extracts were subjected to immunoblot analysis for LC3B and actin. Columns, mean; bars, SD (*N* = 3). ^*∗*^*p* < 0.05; ^*∗∗*^*p* < 0.01.

To determine the role of autophagy in dovitinib-treated breast cells, the present study cotreated with various concentrations of dovitinib and 20 *μ*mole/L autophagy inhibitor, bafilomycin A1, in three breast cancer cells: MCF-7, MDA-MB-231, and MDA-MB-468 cells. The percentage of viable cells increased in the presence of bafilomycin A1 compared to that in the absence of bafilomycin A1. However, bafilomycin A1 exhibited the maximal autophagy inhibiting efficacy on MCF-7 cell line when compared to those in the MDA-MB-231 and MDA-MB-468 cell lines (Figure 3(a)). We further validated the inhibitory effects of bafilomycin A1 on dovitinib-induced activation of LC3B by western blotting. Bafilomycin A1 treatment reduced the accumulation of LC3B by 15 *μ*mol/L dovitinib in MCF-7 breast cancer cells, whereas it caused the increased accumulation of LC3B in MDA-MB-468 cells (Figure 3(b)). Since bafilomycin A1 has been reported to block the fusion of autophagosomes with lysosomes [30], our results interestingly suggest that MCF-7 cells were relatively less activated to autophagosome marker LC3-II compared to MDA-MB-468 cells. As a result, an autophagy inhibitor, bafilomycin A1, blocked dovitinib-induced autophagy in various breast cancer cells, especially MCF-7, and reduced the anti-tumor effects of dovitinib.

### 3.4. Dovitinib Triggered Apoptotic Cell Death in Human Breast Cancer Cells

**Figure 4:**
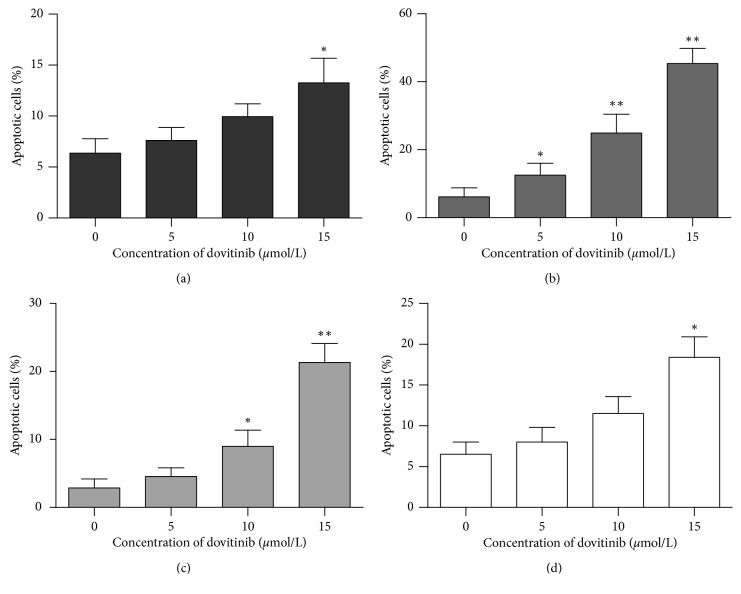
Dovitinib induced apoptosis in breast cancer cells. Human breast cancer cells were exposed to dovitinib at the indicated doses for 24 h. The cells were fixed by EtOH and stained with propidium iodide. Apoptotic cells were measured and determined by flow cytometry. Columns, mean; bars, SD (*N* = 3). ^*∗*^*p* < 0.05; ^*∗∗*^*p* < 0.01. (a) MCF-7. (b) MDA-MB-231. (c) MDA-MB-468. (d) SK-BR-3.

Recent studies reported that dovitinib showed antitumor activity by inhibiting cell proliferation and inducing apoptosis in breast and colorectal cancer cells [31, 32]. However, accumulated studies suggest that autophagy induces chemoresistance against chemotherapeutic agents by inhibiting apoptosis of cancer cells [33]. Our prior finding showed that dovitinib increased autophagy in various breast cancer cells, and the antitumor effect of dovitinib could be restricted by autophagic cell death. To determine whether autophagy is associated with the suppression of dovitinib-induced apoptotic cell death, the nucleic acid stain propidium iodide (PI) flow cytometric assay was used for the evaluation of the number of hypodiploid cells undergoing a late stage of apoptosis process (sub-G1) in the present study. MCF-7, MDA-MB-231, MDA-MB-468, and SK-BR-3 were exposed to dovitinib at the indicated concentration for 24 hours. Dovitinib increased apoptotic cell death in a dose-dependent manner on all tested cell lines. However, the percentages of dovitinib-induced apoptotic cells in 4 human breast cancer cells represented a significant difference. Treating 15 *μ*mole/L dovitinib induced about 15%, 40%, 25%, and 17% cell apoptosis at 24 h for MCF-7, MDA-MB-231, MDA-MB-468, and SK-BR-3 cells, respectively (Figure 4). The data clearly show that increased dovitinib-induced autophagy led to decreased percentages of apoptotic cells on MCF-7 cells, whereas decreased dovitinib-induced autophagy led to increased percentages of apoptotic cells on MDA-MB-468 cells. Thus, we considered that the MCF-7 and MDA-MB-468 cell lines are better comparing the cell model in this experiment to reflect the true dovitinib-mediated apoptotic and autophagic cell death.

**Figure 5:**
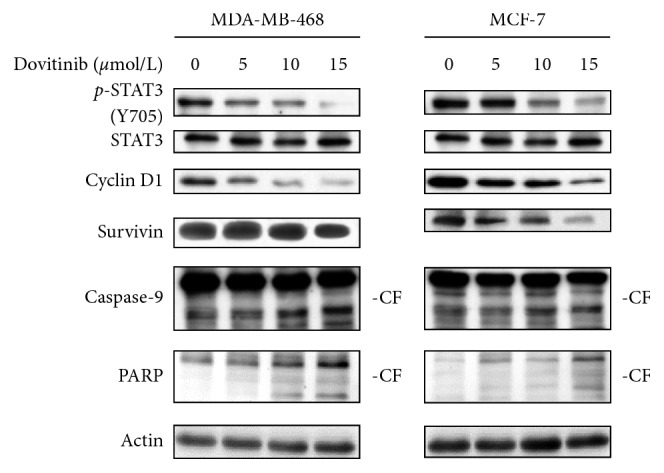
Dovitinib inhibited pSTAT3, apoptotic signaling pathway. Cells were exposed to drugs for 24 h. Cell extracts were subjected to immunoblot analysis for STAT3, pSTAT3, caspase 9, cyclin D1, PARP, survivin, LC3, and actin. CF: cleavage form.

In order to provide a better understanding of the molecular mechanisms underlying dovitinib-induced apoptosis, the detection of apoptotic-related protein expression is required. Incubation with dovitinib using a range of concentrations (5–15 *μ*mole/L) for 24 h resulted in a gradual and dose-dependent decrease in the level of *p*-STAT3^Tyr705^ and the downstream targets activated by STAT3, such as cyclin D1, and survivin in both MCF-7 and MDA-MB- 468 cells, whereas the total STAT3 protein was not influenced (Figure 5). Meanwhile, the protein expression levels of cleaved caspase-9 and cleaved poly (ADP-ribose) polymerase (PARP) were markedly increased by dovitinib in a dose-dependent manner. These observations suggested that dovitinib interferes with STAT3 signaling and downstream targets resulting in apoptosis in both MCF-7 and MDA-MB-468 cell lines.

### 3.5. Overexpression of STAT3 Rescued Dovitinib-Induced Apoptosis in Human Breast Cancer Cells

**Figure 6:**
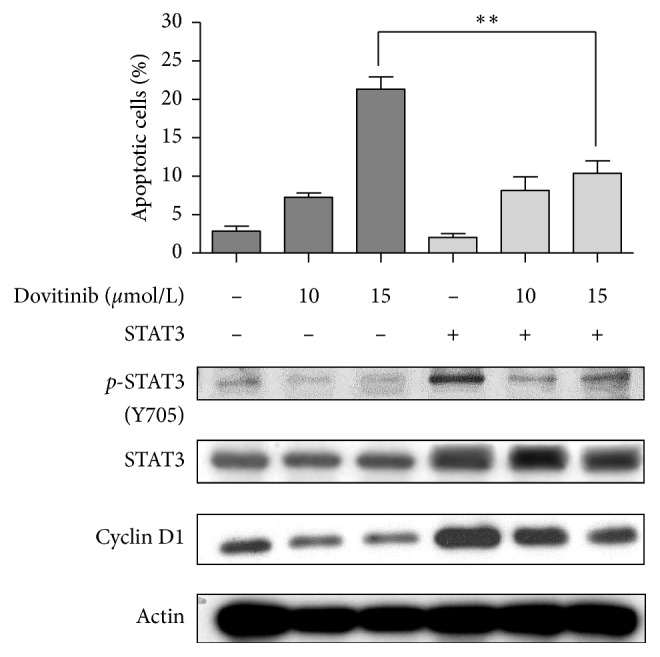
Overexpression of STAT3 rescued breast cancer cells from apoptosis. MDA-MB-468 cells (wild type or STAT3) were treated with dovitinib (10 *μ*mole/L) for 24 h. Apoptotic cells (sub-G1) were analyzed by flow cytometry. Columns, mean; bars, SD (*N* = 3). ^*∗*^*p* < 0.05; ^*∗∗*^*p* < 0.01.

The previous research pointed out that dovitinib downregulates the *p*-STAT3 and subsequently reduced the levels of expression of STAT3-related proteins Mcl-1, survivin, and cyclin D1 in a time-dependent manner in human hepatocellular carcinoma (HCC) [22]. In our present study, the wild and overexpression of STAT3 MDA-MB-468 breast cancer cells were treated with dovitinib for 24 h and cell apoptosis and expression of STAT3/cyclin D1 axis were analyzed subsequently. The results indicated that 10 and 15 *μ*mole/L dovitinib treatments in wild-type MDA-MB-468 cells cause 7.8 ± 1.1% and 20.7 ± 2.8% cell apoptosis, respectively. However, 10 and 15 *μ*mole/L dovitinib induced 8.1 ± 2.6% and 8.9 ± 2.7% apoptotic cells, respectively, in overexpression of STAT3 MDA-MB-468 cells (Figure 6). The ratio of apoptotic cells in overexpression of STAT3 MDA-MB-468 cells significantly reduced after dovitinib treatment compared to that in the wild-type MDA-MB-468 cells. Furthermore, the expression levels of the protein *p*-STAT3, STAT3, and cyclin D1 were markedly increased in overexpression of STAT3 MDA-MB-468 cells following dovitinib treatment at various concentrations compared with the wild-type MDA-MB-468 cells.

**Figure 7:**
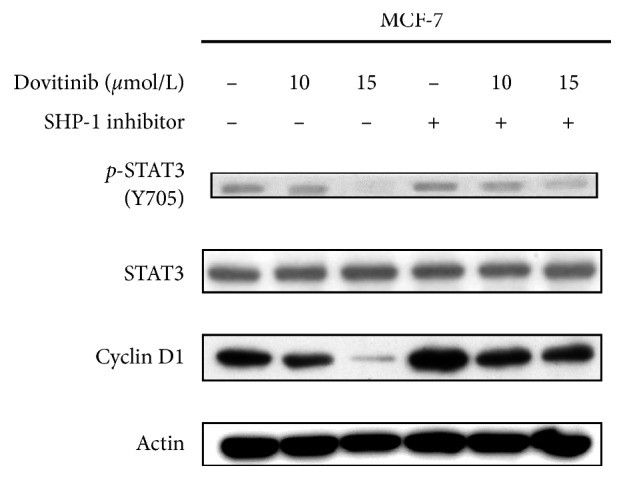
SHP-1 inhibitor decreased the STAT3-inactivating effect of dovitinib. MCF-7 cells were treated with dovitinib (10 and 15 *μ*mole/L) for 24 h. Cell extracts were subjected to immunoblot analysis for STAT3, pSTAT3, cyclin D1, and actin.

Otherwise, since STAT3 has been demonstrated to be a target underlying dovitinib-induced cellular cytotoxicity and apoptosis, we are also interested in that if the other STAT3 negative regulator, SH2-domain-containing phosphatase 1 (SHP-1), is involved in dovitinib-mediated downregulation of STAT3/cyclin D1 axis. SHP-1 is a nonreceptor protein tyrosine phosphatase (PTP) that notably has tumor-suppressive potential due to its negative regulation of STAT3 oncogenic signaling during tumor progression [34, 35]. The present study examined whether blocking SHP-1 affected the downregulation effect of dovitinib in the STAT3/cyclin D1 axis. As shown in Figure 7, the expression levels of *p*-STAT3 and cyclin D1 were decreased in response to dovitinib. However, the expression levels of *p*-STAT3 and cyclin D1 were markedly increased in response to dovitinib and SHP-1 inhibitor cotreatment compared with cells treated with dovitinib alone. Taken together, the SHP-1 inhibitor reversed the dovitinib-induced downregulation of *p*-STAT3, indicating that SHP-1 mediated the STAT3 inhibition effect of dovitinib.

## 4. Discussion

In this study, we showed that dovitinib inactivates STAT3 through SHP-1 to suppress the growth of human breast cancer via induction of both apoptosis and autophagy. Further analysis of the mechanisms discovered that downregulation of STAT3 and cell apoptotic status induced by dovitinib could be reversed by inhibiting the activity of SHP-1, the *p*-STAT3 phosphatase. Moreover, we also disclosed an interesting finding that autophagy was also involved in dovitinib-mediated cell death in human breast cancers. Decreased expression of Mcl-1, the downstream molecule of *p*-STAT3, was responsible for the dovitinib-induced autophagy since its low expression should free beclin-1 and result in the formation of autophagosome [36]. Autophagy induced by dovitinib was confirmed to play as an assassin to attack tumor cells when dovitinib triggered it. These findings suggested that dovitinib could be a potential target therapy reagent for use in treating human breast cancer. In addition, the STAT3-associated molecular events pointed out a more specific application of dovitinib.

Dovitinib has shown a significant antitumor effect on human breast cancer cells. Dovitinib could downregulate *p*-STAT3 and subsequently influence the downstream STAT3-related proteins, such as Mcl-1 [37], PARP [38], cyclin D1 [39], and survivin [40]. These proteins are in charge of several major cellular events, including enhancing cell survival by apoptosis inhibition, DNA repairing, cell cycle progression, and regulating apoptosis [41]. There are many findings to prove the importance of STAT3 signaling in carcinogenesis and have contributed to the designs of new therapeutic targets [42, 43]. It has the ability to control the expression of the antiapoptotic and proliferative gene and also plays a part in creating the tumorigenic microenvironment, which is crucial for tumor progression in several human cancers [44–46]. In human breast cancer, STAT3 is critical in survival and proliferation of tumor-correlated cells, which are tumor-supporting cells [47] and, also, is a promoter in the human breast progression [44] and breast tumor progression [48]. Moreover, STAT3 has been proven to mediate the EGF-stimulating growth and survival effects of human breast cancer cells *in vitro* and, possibly, *in vivo* [49]. Clearly, this evidence indicates STAT3 is constitutively activated in the mammary tumors and contributes to cell transformation, progression, and survival in human breast cancer [50, 51]. Also, several STAT3-related proteins, such as survivin and cyclin D1, are found overexpressed in human breast cancer tissues [52–55]. The complicated involvement of STAT3 and its downstream molecules in cell fate determination has made STAT3 a convincible target in cancer therapy [22, 41, 56].

Dovitinib is a multitarget receptor tyrosine kinase inhibitor and has been reported with inhibition of fibroblast growth factor receptor (FGFR) on metastatic breast cancer patients [17]. Most of the reports about dovitinib are focused on exploring the clinical efficacy in different cancers [57]. There is little research discussing the detailed mechanism of dovitinib in cancer cells. We have shown dovitinib had significant antitumor effects in breast cancer cells with downregulation of *p*-STAT3 and its related molecules to result in cell apoptosis. Being consistent with the previous finding in hepatocellular carcinoma [22], the intrinsic apoptotic pathway (caspase 9) was involved in this dovitinib-mediated tumor cell death.

In addition, we firstly revealed it also caused autophagic cell death in human breast cancer. Autophagy is a vitally catabolic process which involves cell degradation of unneeded or dysfunctional cytosolic components with cooperation to lysosome digestion while cells are under survival stress or starvation [58]. The digested cellular materials will be recycled to maintain cell survival. However, once the cells experienced over or constitutively activated autophagy, the cells would be killed eventually, and this is the so-called autophagic programmed cell death or autophagic death [59]. Because of the dual role of autophagy, it becomes important in the cancer treatments [60–62]. Our data revealed dovitinib not only triggers apoptosis (Figure 4) but also conducts the autophagic death of human breast cancer cells (Figure 3). By simultaneously activating two of these significant cell death machinery, dovitinib could effectively decrease the proliferation of cancer cells.

There were several researches reporting that certain chemotherapy agents would induce autophagy, with pro-survival or pro-death effect [63–66]. One study also declared that autophagy facilitates the resistance to the breast cancer therapeutic agent, trastuzumab [67]. Our results showed dovitinib-induced autophagy to synergize with apoptosis and promote cell death in human breast cancer cells.

Shao et al. [68] also noticed that histone deacetylase (HDAC) inhibitors, both butyrate and suberoylanilide hydroxamic acid (SAHA), can induce apoptosis and caspase-independent autophagic cell death in several human cancer cells. HDAC is overexpressed in many cancers [69–71] and plays a role in transcriptional regulation, protein-DNA interaction, protein-protein interaction, and protein stability [72]. Butyrate and SAHA were designed to target HDAC in Shao's study to find the induction of both apoptosis and autophagy might serve as an efficient anticancer strategy. Dovitinib could induce apoptosis in human breast cancer via regulating survivin. As for the onset of autophagic cell death, Tai et al. noted that antitumor agents could induce the release of beclin-1 from Mcl-1 to induce autophagy [36]. Other reports also demonstrated that the decreased expression of cyclin D1 triggered the start of autophagy [73]. Therefore, it could be inferred that the downexpression of Mcl-1 and cyclin D1 was also involved in autophagic cell death in dovitinib-treated breast cancer cells.

## 5. Conclusions

**Figure 8:**
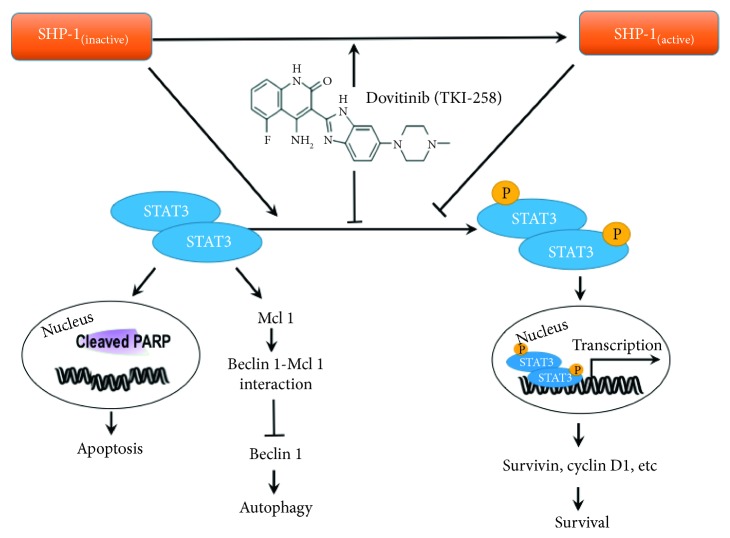
Proposed model of dovitinib-mediated autophagy and apoptosis with regard to the SHP-1/*p*-STAT3 pathway in breast cells. In breast cancer cells, dovitinib inhibit STAT3 phosphorylation and induced autophagy, in part, via releasing of beclin 1 from Mcl-1. Dovitinib also involved in SHP-1 activation and induced apoptotic cell death by downregulating of p-STAT3/cyclin D1 axis and increasing cleaved PARP expression. Accordingly, the present study may provide information regarding the association of autophagy and apoptosis with dovitinib chemotherapy in breast cancer cells, and the regulation of SHP-1/p-STAT3 pathway may be a promising strategy for treating breast cancer cells in response to RTKs inhibitors-based drugs, such as dovitinib.

The present study has proved that dovitinib induced a significant tumor-inhibitory effect through blockade of *p*-STAT3 via SHP-1 activation. Furthermore, the antitumor effects caused by dovitinib were mainly contributed by the activation of programmed cell death that includes both apoptosis and autophagy. The data represented here have provided the evidence for tumor cytotoxic effect of dovitinib to suggest it as a potential target for breast cancer therapy (Figure 8).

## Data Availability

The data used to support the findings of this study are available from the corresponding author upon request.
